# Transition to adult care in young people with neuromuscular disease on non-invasive ventilation

**DOI:** 10.1186/s13052-019-0677-z

**Published:** 2019-07-23

**Authors:** Alessandro Onofri, Hui-Leng Tan, Claudio Cherchi, Martino Pavone, Elisabetta Verrillo, Nicola Ullmann, Maria Beatrice Chiarini Testa, Renato Cutrera

**Affiliations:** 1 Pediatric Pulmonology & Respiratory Intermediate Care Unit, Sleep and Long Term Ventilation Unit, Academic Department of Pediatrics (DPUO), Pediatric Hospital “Bambino Gesù” Research Institute, Piazza S. Onofrio 4, 00165 Rome, Italy; 2 Department of Paediatric Respiratory Medicine, Royal Brompton Hospital, London, 156726 UK

**Keywords:** Neuromuscular disease, Transition to adult care, Long term ventilation, Non-invasive ventilation, Neuromuscular disorders

## Abstract

**Background:**

Long-term mechanical ventilation (LTV) with non-invasive ventilation (NIV) prolongs survival in patients with Neuromuscular Diseases (NMDs). Transition from paediatric to adult healthcare system is an undervalued and challenging issue for children with chronic conditions on mechanical ventilation.

**Methods:**

this retrospective study aims to compare issues of young adults in age to transition to adult care (≥ 15 years old) affected by NMDs on NIV in two different Paediatric Respiratory Units in two different countries: *Bambino Gesù* Children’s Hospital, Research Institute, (Rome, Italy) (BGCH) and the Paediatric Respiratory Unit of the Royal Brompton Hospital (London, UK) (RBHT).

**Results:**

The median (min-max) age at starting ventilation was significantly different in the two groups (16 years old vs 12, *p* = 0.0006). We found significant difference in terms of median age at the time of observation (18 (15–22) vs 17 (15–19) years, *p* = 0.0294) and of type of referral (all the patients from the BGCH group were referred to paediatric services (*n* = 15, 100%), median age 18 (15–22); only 6 patients, in the RBHT group, with a median age 15.50 (15–17) years, were entirely referred to paediatric service). We found different sleep-disordered breathing assessments 6 full Polysomnographies, 7 Cardio-Respiratory Polygraphies and 2 oximetry with capnography (SpO_2_-tcCO_2_) studies in the BCGH group, while all patients of RBHT group were assessed with an SpO_2_-tcCO_2_ study. All patients from both groups underwent multidisciplinary assessment.

**Conclusions:**

In conclusion, patients with NMDs on NIV in age to transition to adult require complex multidisciplinary management: significant efforts are needed to achieve the proper transition to adult care.

## Introduction

Neuromuscular disorders (NMD) comprise a heterogeneous set of diseases which are mostly clinically evident during childhood. Chronic respiratory failure, sleep disordered breathing (SDB) and systemic disorders are the major cause of morbidity and mortality [1, 2]. Since the late 1990s the option of long term mechanical ventilation (LTV) support and the improvements in standards of care are allowing children with NMD to survive throughout childhood [3–5].

Both non-invasive ventilation (NIV) and invasive mechanical ventilation (IMV) are therapeutic options that can prolong survival in patients with severe muscular weakness. Provision of long term non-invasive ventilation support and the population of children who survive reaching adulthood are exponentially increasing [6, 7].

New emerging genetic and molecular therapies are extending survival of children with NMD as well as the better management of complications. The increase of life expectancy is having new and unexpected consequences. These changes in natural history are accompanied by several new issues that have never been dealt with. Smooth transition to adult care is certainly one of the most challenging.

The transition from paediatric to adult healthcare system, defined by Blum as “the purposeful, planned movement of adolescents with chronic physical and medical conditions from child-centered to adult-oriented healthcare” [8], is one of the most undervalued and challenging issues for children with NMD. The lack of standardized programs has been pointed out in the literature and has been recognized as an important issue for children with chronic diseases [9].

The recent consensus on standards of care in spinal muscular atrophy (SMA) acknowledged that transition is a challenging period for many patients with milder forms of the disease and needs further attention [10].

Most commonly used clinical care recommendations on Duchenne muscular dystrophy (DMD) management do not include any focus about transition to adult care services despite life expectancy of these patients having increased over the years [11].

Some countries have made efforts to support transitional care with policy documents. Some other countries have no regulation at all, using local rules without any formal plan for transition. There is a lack of attention by different jurisdictions and little data in the literature about issues of young adults affected by NMD on NIV who are of age to transition to adult services. Moreover, there is little information about the best clinical practices and management of these patients during this period.

This study aims to provide a retrospective comparison between two different Paediatric Respiratory Units in tertiary care hospitals, in two different countries, that are dealing with young adult patients affected by NMD on NIV, focusing on their main issues. We provided a retrospective analysis of patients affected by NMD, evaluated in the last three years (2014–2016), on NIV and in the age of transition to adult care in two institutions: the Respiratory Unit in the Royal Brompton Hospital (London, UK) (RBHT) where a transition programme starting from the age of 15 years old is in place, and the Respiratory Unit *Bambino Gesù* Children Hospital, Research Institute (Rome, Italy) (BGCH), where there isn’t any transition plan.

## Material and methods

In this retrospective case notes review we examined the databases of the Paediatric Respiratory Care Unit *Bambino Gesù* Children’s Hospital, Research Institute (Rome, Italy) and of the Royal Brompton Hospital (London, UK). All the patients were regularly followed by both Paediatric Respiratory Care Units, with scheduled admissions to titrate NIV and assess SDB with sleep studies.

We determined the number of patients aged equal or older than 15 years who:

- Had a confirmed diagnosis of neuromuscular disease;
- Were on long-term ventilation (LTV) with NIV support (for at least 3 months);
- Were of the age to start transition to adult care services.

We chose the age of 15 years old as the cut-off age because in RBHT, the transition program starts from 15 years of age and above.

All data were obtained from clinical notes and no identifiable patient data were included. The study was conducted from the 1st of January 2014 to the 31st of December 2016. We excluded from our analysis patients whose data were incomplete or lost during the follow up. Moreover, we excluded patients with an uncertain or on-going diagnosis and patients on LTV with IMV.

The categories of information collected are listed in Table 1.

**Table 1:** Summary of information gathered

● Date of birth	
● Sex	
● Ethnicity	
● Date of evaluation	
● BMI	
● Diagnosis	
● Overall admissions and admissions for sleep assessment during the last 12 months	
● Acute respiratory exacerbations during the last 12 months	
● Chronic colonization (specify pathogens)	
● Antibiotic prophylaxis	
● Feeding	
● Physiotherapy: N physio session/week, use of physio aids (cough assistance, VEST, others)	
● Pulmonary function evaluation: FVC Z score	
● Age at initiation NIV	
● Last sleep study: ODI 4%, mean SpO_2,_ mean CO_2_%, time overnight spent CO_2_ > 6.5 kPa, % time overnight spent SpO_2_ < 90%, AHI	
● Time passed since previous sleep study (months)	
● Mode of ventilation: and main NIV settings (IPAP, EPAP)	
● Interface mask	
● Time passed depending on ventilator assistance	
● Multidisciplinary assessment	
● Comorbidities	

*BMI* Body Mass Index, *VEST* VEST airway clearance system, *FVC* Forced Vital Capacity, *ODI* Oxygen Desaturation Index, *AHI* Apnea-Hypopnea Index, *IPAP* Inspiratory Positive Airway Pressure, *EPAP* Expiratory Positive Airway Pressure

## Statistical ANALYS

We used Microsoft Excel 2016 to create a database and Graphpad Prisma (version 7.03) to analyse data. Data were expressed as median (range). Recorded variables were compared using the t student test for unpaired data. We used parametric testing when data assumed a Gaussian distribution, and nonparametric testing when data were not normally distributed. A *p* value < 0.05 was considered statistically significant.

Ethical approval in both hospitals was not required for this retrospective evaluation as all data were anonymised and was part of service evaluation.

## Results

### Overall patient characteristics

We analysed the medical records of 18 patients from the Paediatric Respiratory Unit of the *Bambino Gesù* Children’s Hospital, Research Institute (Rome, Italy) (BGCH) and 17 patients from the Paediatric Respiratory Unit of the Royal Brompton Hospital (London, UK) (RBHT). All records were cross referenced with the electronic patient records in both groups. Three patients from the first group were excluded: two of them had incomplete data, one was lost to follow-up. Two patients from the second group were excluded because their data were incomplete.

In the BGCH group we finally selected 15 children with the following underlying neuromuscular diseases: DMD (10, 67%), SMA type II (3, 20%), and congenital myopathies (2, 13%). In the RBHT group we finally selected: SMA type II (7, 47%), congenital muscular dystrophies (5, 33%) and congenital myopathies (3, 20%).

The median age at the time of observation was significantly different in the two groups: 18 (15–22) vs 17 (15–19) years, *p* = 0.0294. Table 2 shows the overall patient characteristics.

**Table 2:** Comparison between the two groups in terms of general features, admissions and exacerbations during the last 12 months

Overall patient characteristics	BGCH	RBHT	*p* value
Age, years^a^	18 (15–22)	17 (15–19)	*p = 0.0294*
Ethnicity			
White n (%)	15 (100%)	7 (47%)	
Asian n (%)	0	7 (47%)	
Black n (%)	0	1 (6%)	
BMI, kg/m^2a^	18.71 (9.1–28.52)	16.45 (11.2–25.8)	*p* = 0.2846
FVC, Z score^a^	−6.24 (− 8.03- -3.17)	−6.93 (− 8.75- -3.89)	*p = 0.0441*
Age at initiation NIV, years^a^	16 (15–20)	12 (5–18)	*p = 0.0006*
Months on follow-up^a^	32 (3–158)	122 (44–162)	*p < 0.0001*
(years)	(2)	(10)	
Months on ventilation^a^	13 (3–67)	51 (3–159)	*p = 0.0198*
(years)	(1)	(4)	
Overall admissions/patient during the last 12 months^a^, n	1 (0–3)	1 (0–4)	*p* = 0.7025
Admissions for sleep assessment/patient during the last 12 months^a^, n	1 (0–3)	1 (0–3)	*p* = 0.8277
Acute exacerbations during the last 12 months^a^, n	0 (0–1)	0 (0–2)	*p* = 0.3143

^a^Median (minimum-maximum)

*BMI* Body Mass Index, *FVC* Forced Vital Capacity, (BGCH = Paediatric Respiratory Unit of the *Bambino Gesù* Children Hospital; RBHT = Paediatric Respiratory Unit of the Royal Brompton Hospital)

### Transition to adult care

All the patients from the BGCH group were cared for by paediatric services (*n* = 15, 100%), median age 18 (15–22).

In the RBHT group, 6 patients with a median age 15.50 (15–17) years, were entirely cared for by paediatric service. Four patients were completing the transition from paediatric to adult service, median age 17 (16–19) years. Five patients were entirely cared for by adult service, median age 18 (17–19) years. All the patients who transitioned to the adult service had completed the process within the 12 months before our analysis.

### Sleep disordered breathing (SDB) assessment

In the BGCH group, 6 patients underwent a full Polysomnography (PSG), 7 patients were assessed with a Cardio-Respiratory Polygraphy (CR-Poly) and 2 patients were assessed with a coupled transcutaneous monitoring of SpO_2−_CO_2_ (SpO_2_-tcCO_2_ study). All patients from the RBHT group were assessed with an SpO_2_-tcCO_2_ study. Table 3 shows the results of the last sleep study in the two groups carried out to follow-up SDB. All the patients of both groups have undergone the sleep study on NIV.

**Table 3:** Comparison of last sleep study results between the two groups and time since the previous sleep study was performed in the referral hospital

Last sleep study results.	BGCH ^a^	RBHT^a^	*p* value
Mean CO_2_ (n, KPas)	5.27 (4.6–6.3)	5.89 (4.16–7.89)	*p* = 0.0596
Time spent CO_2_ > 6.5 KPas (%)	0 (0–35)	1 (0–100)	*p* = 0.1715
Mean SpO_2_ (%)	97 (94–99)	97 (94–99)	*p* = 0.8178
Time spent SpO_2_ < 90% (%)	0 (0–14.2)	0 (0–0.59)	*p* = 0.3374
ODI 4% (events/h)	0.80 (0–6.5)	2.75 (0–10)	*p* = 0.0757
AHI	0.5 (0–5.3)	–	–
Time since last sleep study (months)	7 (1–13)	8 (1–13)	*p* = 0.6851

^a^Median (minimum-maximum)

*ODI* Oxygen Desaturation Index, *AHI* Apnea-Hypopnea Index. (BGCH = Paediatric Respiratory Unit of the *Bambino Gesù* Children Hospital; RBHT = Paediatric Respiratory Unit of the Royal Brompton Hospital)

### Non-invasive ventilation settings

Table 4 summarizes NIV settings in the two groups. BGCH group and RBHT group didn’t show any significant difference in terms of median IPAP 14 (8–21) vs 18 (10–25) cmH_2_O, *p* value = 0.0623. They showed significant difference in the median EPAP setting: 4 (4, 5) vs 5 (4–7) cmH_2_O, p value = 0.0021.

**Table 4:** Comparison of NIV settings in the two groups. PSV = Pressure Support Ventilation. VAPS=Volume Assured Pressure Support

NIV features	BGCH	RBHT
Mode of ventilation		
PSV n (%)	12 (80%)	15 (100%)
VAPS n (%)	3 (20%)	0
Interface		
Nasal mask n (%)	10 (66%)	7 (47%)
Nasal pillows n (%)	3 (20%)	2 (13%)
Full-face mask n (%)	2 (14%)	6 (40%)
Daytime ventilator use		
Nocturnal n (%)	14 (93%)	13 (86%)
12–20 h, n (%)	1 (7%)	2 (14%)
24 h, n (%)	0	0

(BGCH = Paediatric Respiratory Unit of the *Bambino Gesù* Children Hospital; RBHT = Paediatric Respiratory Unit of the Royal Brompton Hospital)

### Physiotherapy and chronic colonization

Thirteen patients (86%) from the BGCH group and eight patients (53%) from the RBHT group were practicing a chest physiotherapy protocol. In the BGCH group, one patient performed chest physiotherapy only when unwell; one other patient had voluntarily stopped any specific chest physiotherapy protocol. Seven patients from the RBHT group (47%) had physiotherapy sessions only when unwell. In both groups, the cough assist machine was used by 7 patients (47%). One patient in the RBHT group used a VEST airway clearance system.

In three patients (20%) of the BGCH group the cough swab was positive for chronic colonization. All three patients were colonized by *Staphylococcus aureus*, one was also colonized with *Klebsiella pneumoniae*, and one also with *Candida albicans*. Eight patients (53%) were not tested for chronic colonization. Four patients (27%) had a negative cough swab test. None of them was on treatment with antibiotic prophylaxis until the last admission. In the RBHT group one patient (7%) had a positive cough swab for *Staphylococcus aureus.* Eight patients had a negative cough swab (53%) and six patients (40%) were not tested. Four patients (27%) were on antibiotic prophylaxis.

### Comorbidities, multidisciplinary assessment and feeding

All patients from both centres underwent multidisciplinary assessment. All patients from the BGCH group were evaluated by different specialists during follow up admissions. They were mostly evaluated by: physiotherapists and cardiologists (100%), neuromuscular disorders specialists (86%), dieticians (47%), psychologists (47%), neuropsychiatrists, endocrinologists, orthopaedics (20%) and nephrologists (13%).

In the RBHT the multidisciplinary assessment was mainly respiratory. All the patients had speech and language therapist (SALT), physiotherapist and dietician assessments in clinic. Two patients had a recorded neuropsychiatric assessment (13%); one patient had an endocrine assessment (7%) and one a urogenital assessment (7%). We had records of seven cardiological referrals (46%). However, data about multidisciplinary assessment in RBHT group might be inaccurate because specialist referrals are made outside to different hospitals and might not be recorded in clinical notes in RBHT.

Among the patients of the BGCH group, only one (7%) had enteral feeding via percutaneous endoscopic gastrostomy (PEG). In the RBHT group eight patients (53%) had enteral feeding via PEG. One of them (7%) received both oral and enteral feeding. Six patients (40%) had oral feeding.

## Discussion

This retrospective case note review reports two different experiences in two different countries, with different transition guidance, highlighting the issues of adolescents with NMD on NIV who are of age to transition to adult care.

*Bambino Gesù* Children’s Hospital does not have any programme of transition from paediatric to adult services. Most of the patients after 18 years old continue to be followed-up by the Paediatric Respiratory team. All the patients have periodic scheduled follow-up admissions to assess SDB, nutrition, physiotherapy plan and the other entire specialist referrals are performed during the admission. In Royal Brompton Hospital all the patients have periodic scheduled follow-up admissions only for assessing SDB with a sleep study. Transition programme usually consists of a first step made when the patient is 15 years old or more; a letter is sent by the paediatric respiratory physician as first referral to adult services. The second step is a joint clinic between paediatric and adult respiratory physicians. The last step is the final clinic visit in the adult department and the first sleep study in the adult Sleep and Ventilation Unit. It is important to note that RBHT has a unique model of care where patients potentially could be followed up from birth to old age. Therefore, transition is within the same hospital from paediatric to adult services. This allows removing one of the main obstacles to good transition that is the lack of communication between the adult and paediatric services. The structure of RBHT allows adult services to take over young adults when they are almost 17 years old. This specific feature could explain our findings of different median age at the time of the observation between the two groups: 18 (15–22) vs 17 (15–19) (*p* = 0.0294). Moreover, 17 years old patients in RBHT have fully or partially left paediatric services, while they are continuing follow-up in paediatric service in BGCH. The effect of this difference could have economic, psychological, organisational implications and requires further prospective evaluation.

Previous studies on NMD have emphasized that NIV is effective at correcting nocturnal hypoventilation and usually well tolerated. NIV can help prevent respiratory complications and prolong life. The overall survival for neuromuscular patients on NIV is high, nevertheless the rate of weaning is low [12–14]. In Italy in 2011 Racca et al. reported 378 children requiring LTV. Patients affected by NMD were a large proportion (187 patients, 49.5%). Among them, the use of NIV was preferred when feasible (61%), especially for patients older than 11 [15]. These numbers are confirmed by Austrian (44% of 143 patients had NMD, and 35.7% on LTV were between 12 and 18 years old) [16] and Swiss surveys (41% of 32 patients had NMD) [17]. Chatwin et al. reported that 40% (181 patients) of their cohort (496 children) on LTV with NIV have transitioned to adult services, with a high prevalence of patients affected by NMD surviving after sixteen years of age [18]. We found significantly different age at starting NIV between the two groups: in the BGCH group the median age was 16, while in the RBHT group, patients started NIV earlier (12 years old). We can explain this difference considering the different underlying diagnosis and the different respiratory functional impairment between the two groups: more DMD in the BGCH group compared to more SMA type II in the RBHT group and different FVC Z score (Forced Vital Capacity) (− 6.24 vs − 6.93, *p* = 0.0441). As already stated in the literature, DMD patients start requiring mechanical ventilation support later than other neuromuscular patients [18].

In terms of respiratory management, British Thoracic Society guidelines recommend at least one annual assessment for sleep-disordered breathing in: a) children who have a vital capacity of < 60% or who have symptoms of sleep apnoea/nocturnal hypoventilation or loss of ambulation because of progressive weakness; b) children who never attain the ability to walk; c) children with diaphragmatic weakness [19]. The 2004 ATS Consensus Statement suggested annual evaluations for SDB by PSG or, if this is not possible, by overnight pulse-oximetry recording with continuous CO_2_ monitoring [20]. Polysomnography is expensive and not always feasible, requiring overnight admission. The overnight pulse-oximetry recording with continuous CO_2_ monitoring showed good results in screening patients with overnight hypoventilation. The two groups under our analysis underwent different overnight sleep monitoring and did not show significant impairment in terms of gas exchange on NIV. All the patients of the RBHT group were assessed by SpO_2_-tcCO_2_ study while the majority of the BGCH group patients underwent CR-Poly or full PSG.

We recognise that our samples are too small to conclude which level of sleep assessment is necessary to monitor patients with NMD on NIV. However, all the patients of both groups showed good gas exchanges during the night, without any hypoventilation on NIV. All the patients of the BGCH group that have received polygraphy or full PSG didn’t show any increase in Apnea-Hypopnea Index (AHI). Our results suggest that overnight pulse-oximetry recording with continuous CO_2_ monitoring could be sufficient to monitor patients with NMD on NIV, performing full PSG or sleep polygraphy only in selected cases and to assess SDB for the first time, if feasible. Further studies are needed to evaluate which approach is more cost-effective. Timing of follow-up sleep studies in both groups was similar (7–8 months).

We considered patients with milder forms of NMD, not requiring tracheostomy, with NIV support since early adolescence or shortly before. Our patients did not show significant hypoventilation during the night with non-invasive ventilation support. Almost all patients used NIV mainly during the night. Non-invasive ventilation was delivered via nasal masks, nasal pillows, oro-nasal masks or full-face masks. We observed a preferential use of nasal mask and nasal pillows when feasible, in both groups. As regards the ventilatory settings, the main difference in the two groups was in the use of EPAP, which was significantly higher in RBHT patients. In the Italian group, in a small number of cases, a target volume setting was used, while in the English group all the patients were using PSV S/T mode. All this data obviously could be affected by the different diagnoses and the worse lung function impairment of the RBHT rather than BGCH group. The limited number of patients does not allow a wider consideration about trends in ventilator mode usage in the two countries.

We explored the other main requirements of these patients other than mechanical ventilation features. Young adults with NMD often require intensive chest physiotherapy plans and both groups have similar number of physiotherapy sessions/week albeit more patients in the RBHT do physiotherapy only when unwell (increased secretions, acute exacerbations, increased work of breathing or dyspnoea). The majority of the patients from both centres have a specific physiotherapy plan involving mostly respiratory and neuromotor rehabilitation, manual cough assistance, use of cough assist machine and use of air stacking techniques. Cough assistance is commonly used in patients with severe muscular weakness and low cough peak flow [21]; the domiciliary usage of this aid is the same in the two groups.

The prevention of respiratory infections is optimised with the proper use of airway clearance techniques. The use of antibiotic prophylaxis is more widespread in the UK than in other countries such as Italy and our data confirms this trend. However, at the moment, evidence that antibiotic prophylaxis in certain groups of high-risk children can reduce pneumonia, exacerbations, hospital admission and mortality is inconclusive [22]. Our two groups had few patients with chronic colonization, the most frequent pathogen was *Staphylococcus aureus*.

A multidisciplinary approach is required for patients with NMD. A skilled multidisciplinary team is important not only in managing comorbidities but also to engage patients and parents in the decision-making process during the natural history of the disease [23]. Multidisciplinary approach is likely to fragment during transition from paediatric to adult care. In the two institutions, we found different approaches. In BGCH, the multidisciplinary assessment is done in the Respiratory Care Unit during scheduled admissions for SDB assessment. It consists most frequently of cardiologists, neuromuscular disorders specialists, dieticians, psychologists. Any other required referral is made during the admission. RBHT is specialist hostpital for the treatment and management of lung and heart disease in paediatric and adult patients. The Neuromuscular consultant is usually outside the hospital. Inside the hospital physiotherapy, dietician and Speech and Language Therapist assessments are guaranteed during clinic appointments or during admissions for follow-up sleep study.

Our results in the two groups are consistent with the main findings in the literature in terms of comorbidities. Scoliosis is the most common comorbidity in patients affected by neuromuscular disorders. The spinal deformity usually rapidly progresses during growth: bracing or spinal surgery when feasible are the first line therapies [24]. Dilated cardiomyopathy is one of the most serious and often fatal complications of DMD [25]. Swallowing difficulties, nutrition issues and gastrointestinal dysmotility are more frequent in SMA patients [11]. As reported in the literature in patients with DMD with prolonged survival, nephrolithiasis is associated in patients affected by SMA or DMD [26]. Bone management is an important issue for young adult patients with NMD. Lack of ambulation, decreased mechanical loading forces are risk factors for poor bone mineral density (BMD) and increased risk of fractures. The use of corticosteroids for long-term treatment is certainly another factor that could decrease BMD. Endocrine assessment and evaluation of BMD is required to identify effective interventions to prevent osteoporosis and fractures and the initiation of treatments to increase mineral bone density such as bisphosphonates, vitamin D and calcium supplement [27, 28].

Adolescence is a difficult time for young adults with disability. In both groups, psychological comorbidity was present. Several patients were under treatment for anxiety and depression and our data confirm that mental health requires special consideration in adolescents with NMD [29]. The psychosocial domain should be carefully considered in future guidelines especially considering psychological issues of adolescence in patients with chronic disability. It is crucial to consider the need for a smooth process of transition to avoid undue psychological burdens for these patients and to involve them as much as possible in occupational and leisure activities.

Our study has several limitations as it was retrospective and we excluded unusable or missing data. The samples are small, different in terms of underlying diagnosis, lung function and age. It is important however that our data show the different management strategies in two different countries highlighting the differences and similarities. Further prospective multicentre studies are needed to deepen our understanding and to provide a more comprehensive analysis.

## Conclusions

Our retrospective analysis shows that patients with NMD on NIV require complex management in terms of multidisciplinary assessment, SDB evaluation, monitoring of NIV settings, prevention of respiratory infections, psychological and nutritional issues. Therefore, it is important to develop cost effective models to guarantee that these aspects all come together, without any deficiency during the process of transition. More international survey data are needed to evaluate the burden of NMD adolescent patients on respiratory support in different countries. Further prospective studies are needed to evaluate the psychological and economic impact of having (or not) a transition program in young adults with NMD. In conclusion, providing a smooth transition from paediatric to adult services is a very challenging task for jurisdictions, administrations, adult physicians and paediatricians but is imperative for patients and their families.

## Data Availability

The datasets used and/or analysed during the current study are available from the corresponding author on reasonable request.

## Abbreviations

BGCH: Bambino Gesù Children’s Hospital, Research Institute, (Rome, Italy)
CR-Poly: Cardio-Respiratory Polygraphy
DMD: Duchenne Muscular Atrophy
EPAP: Expiraytory Positive Airway Pressure
IMV: Invasive Mechanical Ventilation
IPAP: Inspiratory Positive Airway Pressure
LTV: Long Term Ventilation
NIV: Non-Invasive Ventilation
NMDs: Neuromuscular diseases
PEG: Percutaneous Endoscopic Gastrostomy
PSG: Polysomnography
RBHT: Paediatric Respiratory Unit of the Royal Brompton Hospital (London, UK)
SDB: Sleep Disordered Breathing
SMA: spinal muscular atrophy
SpO_2_-tcCO_2_ studies: oximetry with capnography studies

## References

[1] Howard RS, Wiles CM, Hirsch NP (1993). Respiratory involvement in primary muscle disorders:assessment and management. Q J Med.

[2] Perrin C, Unterborn JN, Ambrosio CD (2004). Pulmonary complications of chronic neuromuscular diseases and their management. Muscle Nerve.

[3] Eagle M, Bourke J, Bullock R, Gibson M, Mehta J, Giddings D, Straub V, Bushby K (2007). Managing Duchenne muscular dystrophy--the additive effect of spinal surgery and home nocturnal ventilation in improving survival. Neuromuscul Disord.

[4] Flanigan KM (2014). Duchenne and Becker muscular dystrophies. Neurol Clin.

[5] Farrar MA, Vucic S, Johnston HM, du Sart D, Kiernan MC (2013). Pathophysiological insights derived by natural history and motor function of spinal muscular atrophy. J Pediatr Orthop.

[6] Gregoretti C., Ottonello G., Chiarini Testa M. B., Mastella C., Rava L., Bignamini E., Veljkovic A., Cutrera R. (2013). Survival of Patients With Spinal Muscular Atrophy Type 1. PEDIATRICS.

[7] Simonds AK (2003). Home ventilation. Eur Respir J Suppl.

[8] Blum RW (1995). Transition to adult health care: setting the stage. J Adolesc Health.

[9] Agarwal A, Willis D, Tang X, Bauer M, Berlinski A, Com G, Ward WL, Carroll JL (2015). Transition of respiratory technology dependent patients from pediatric to adult pulmonology care. Pediatr Pulmonol.

[10] Finkel Richard S., Sejersen Thomas, Mercuri Eugenio, Bertini E., Chen K., Crawford T.O., Dubowitz V., de Lemus M., Graham R., Hurst Davis R., Iannaccone S., Kirschner J., Main M., Mayer O., Mazzone E., Montes J., Muntoni F., Murphy A., Quijano-Roy S., Robertson A., Schroth M., Simonds A., Snyder B., Vitale M., Wittchen A., Woods S., Qian Y., Wirth B. (2017). 218th ENMC International Workshop. Neuromuscular Disorders.

[11] Bushby K, Finkel R, Birnkrant DJ, Case LE, Clemens PR, Cripe L, Kaul A, Kinnett K, McDonald C, Pandya S, Poysky J, Shapiro F, Tomezsko J, Constantin C (2010). DMD care considerations working group. Diagnosis and management of Duchenne muscular dystrophy, part 1: diagnosis, and pharmacological and psychosocial management. Lancet Neurol.

[12] Simonds AK (2006). Recent advances in respiratory care for neuromuscular disease. Chest.

[13] McDougall CM, Adderley RJ, Wensley DF, Seear MD (2013). Long-term ventilation in children: longitudinal trends and outcomes. Arch Dis Child.

[14] Bushby Katharine, Finkel Richard, Birnkrant David J, Case Laura E, Clemens Paula R, Cripe Linda, Kaul Ajay, Kinnett Kathi, McDonald Craig, Pandya Shree, Poysky James, Shapiro Frederic, Tomezsko Jean, Constantin Carolyn (2010). Diagnosis and management of Duchenne muscular dystrophy, part 1: diagnosis, and pharmacological and psychosocial management. The Lancet Neurology.

[15] Racca F, Berta G, Sequi M (2011). Long-term home ventilation of children in Italy: a National Survey. Pediatr Pulmonol.

[16] Weiss S, Van Egmond-Fröhlich A, Hofer N, Pfleger A, Rath R, Schwarz R, Kurz H, Waibel V, Kenzian H, Kommer E, Wadlegger F, Stelzl W, Keck B, Grigorow I, Kerbl R, Sauseng W, Frischer T, Eber E, Bernert G (2016). Long-term respiratory support for children and adolescents in Austria: a National Survey. Klin Padiatr.

[17] Kamm M, Burger R, Rimensberger P, Knoblauch A, Hammer J (2001). Survey of children supported by long-term mechanical ventilation in Switzerland. Swiss Med Wkly.

[18] Chatwin M, Tan HL, Bush A, Rosenthal M, Simonds AK (2015). Long term non-invasive ventilation in children: impact on survival and transition to adult care. PLoS One.

[19] Hull J, Aniapravan R, Chan E, Chatwin M, Forton J, Gallagher J, Gibson N, Gordon J, Hughes I, McCulloch R, Russell RR, Simonds A (2012). British Thoracic Society guideline for respiratory management of children with neuromuscular weakness. Thorax..

[20] Finder JD, Birnkrant D, Carl J, Farber HJ, Gozal D, Iannaccone ST, Kovesi T, Kravitz RM, Panitch H, Schramm C, Schroth M, Sharma G, Sievers L, Silvestri JM, Sterni L (2004). American Thoracic Society. Respiratory care of the patient with Duchenne muscular dystrophy: ATS consensus statement. Am J Respir Crit Care Med.

[21] Chatwin M, Ross E, Hart N, Nickol AH, Polkey MI, Simonds AK (2003). Cough augmentation with mechanical insufflation/exsufflation in patients with neuromuscular weakness. Eur Respir J.

[22] Onakpoya IJ, Hayward G, Heneghan CJ. Antibiotics for preventing lower respiratory tract infections in high-risk children aged 12 years and under. Cochrane Database Syst Rev. 2015;(9):CD011530. 10.1002/14651858.CD011530.pub2 PMC10624245 26408070

[23] Bushby K, Finkel R, Birnkrant DJ (2010). Diagnosis and management of Duchenne muscular dystrophy, part 2: implementation of multidisciplinary care. Lancet Neurol.

[24] Halawi MJ, Lark RK, Fitch RD (2015). Neuromuscular scoliosis: current concepts. Orthopedics..

[25] Shirokova N, Niggli E (2013). Cardiac phenotype of Duchenne muscular dystrophy: insights from cellular studies. J Mol Cell Cardiol.

[26] Shumyatcher Y, Shah TA, Noritz GH, Brouhard BH, Spirnak JP, Birnkrant DJ (2008). Symptomatic nephrolithiasis in prolonged survivors of Duchenne muscular dystrophy. Neuromuscul Disord.

[27] Bell JM, Shields MD, Watters J, Hamilton A, Beringer T, Elliott M, Quinlivan R, Tirupathi S, Blackwood B (2017). Interventions to prevent and treat corticosteroid-induced osteoporosis and prevent osteoporotic fractures in Duchenne muscular dystrophy. Cochrane Database Syst Rev.

[28] Wasserman HM, Hornung LN, Stenger PJ, Rutter MM, Wong BL, Rybalsky I, Khoury JC, Kalkwarf HJ (2017). Low bone mineral density and fractures are highly prevalent in pediatric patients with spinal muscular atrophy regardless of disease severity. Neuromuscul Disord.

[29] Latimer R, Street N, Conway KC, James K, Cunniff C, Oleszek J, Fox D, Ciafaloni E, Westfield C, Paramsothy P (2017). Muscular dystrophy surveillance, tracking, and research network (MD STAR net). Secondary conditions among males with Duchenne or Becker muscular dystrophy. J Child Neurol.

